# AT 1 inhibition mediated neuroprotection after experimental traumatic brain injury is dependent on neutrophils in male mice

**DOI:** 10.1038/s41598-023-33797-6

**Published:** 2023-05-07

**Authors:** Ralph Timaru-Kast, Shila P. Coronel-Castello, Tobias J. Krämer, André V. Hugonnet, Michael K. E. Schäfer, Anne Sebastiani, Serge C. Thal

**Affiliations:** 1 Department of Anesthesiology, University Medical Center of the Johannes Gutenberg-University, Langenbeckstrasse 1, 55131 Mainz, Germany; 2 Focus Program Translational Neuroscience (FTN), University Medical Center of the Johannes Gutenberg-University, Langenbeckstrasse 1, 55131 Mainz, Germany; 3 Faculty of Health, University of Witten/Herdecke, Alfred-Herrhausen-Strasse 50, 58455 Witten, Germany; 4 Department of Anesthesiology, HELIOS University Hospital Wuppertal, University of Witten/Herdecke, Heusnerstrasse 40, 42283 Wuppertal, Germany

**Keywords:** Brain injuries, Experimental models of disease, Preclinical research, Trauma, Granulocytes, Neuroimmunology, Regeneration and repair in the nervous system

## Abstract

After traumatic brain injury (TBI) cerebral inflammation with invasion of neutrophils and lymphocytes is a crucial factor in the process of secondary brain damage. In TBI the intrinsic renin-angiotensin system is an important mediator of cerebral inflammation, as inhibition of the angiotensin II receptor type 1 (AT1) reduces secondary brain damage and the invasion of neutrophil granulocytes into injured cerebral tissue. The current study explored the involvement of immune cells in neuroprotection mediated by AT1 inhibition following experimental TBI. Four different cohorts of male mice were examined, investigating the effects of neutropenia (anti-Ly6G antibody mediated neutrophil depletion; C57BL/6), lymphopenia (RAG1 deficiency, RAG1^−/−^), and their combination with candesartan-mediated AT1 inhibition. The present results showed that reduction of neutrophils and lymphocytes, as well as AT1 inhibition in wild type and RAG1^−/−^ mice, reduced brain damage and neuroinflammation after TBI. However, in neutropenic mice, candesartan did not have an effect. Interestingly, AT1 inhibition was found to be neuroprotective in RAG1^−/−^ mice but not in neutropenic mice. The findings suggest that AT1 inhibition may exert neuroprotection by reducing the inflammation caused by neutrophils, ultimately leading to a decrease in their invasion into cerebral tissue.

## Introduction

The leading cause of trauma-related death and profound disability in developed countries is traumatic brain injury (TBI)^1^. After the mechanical impact (primary injury), deleterious mechanisms such as cerebral inflammation trigger the propagation of a secondary brain injury into the adjacent healthy tissue^2^. The early and vigorous immune response is marked by the activation of the innate immunity, which involves brain resident microglia, and infiltration of peripheral immune cells, particularly neutrophils, into the injured tissue^3,4^. The subsequent engagement of the adaptive immune system is characterized by the invasion of B and T lymphocytes^3–6^. One crucial mediator of neuroinflammation is angiotensin II (AngII)^4,7,8^, the main effector peptide of the intrinsic cerebral renin-angiotensin system (RAS). The activation of AngII receptor type 1 (AT1) mediates vasoconstriction and various pro-inflammatory processes, aggravating secondary brain damage following cerebral insults^8–14^. AT1 signaling is mediated by extracellular signal-regulated kinases (ERK1/2), mitogen-activated protein kinases (MAPK, JNK, p38MAPK), glycogen synthase kinase, Rho/ROCK kinase, receptor tyrosine- (PDGF and EGFR) and non-receptor tyrosine-kinases (Src, Pyk2, and JAK/STAT)^15^. Recent experimental TBI studies have indicated that AT1 inhibition reduces secondary brain damage and improves neurological outcome^4,8,16,17^. In mice treated with the specific AT1 inhibitor candesartan, we observed reduced microglial activation and a decrease in the infiltration of neutrophils into injured brain tissue^4^. These findings suggest that one possible underlying mechanism of AT1 inhibition-mediated neuroprotection may be a reduction in neutrophil invasion^4^. In the current study, our aim was to test this hypothesis by investigating the role of neutrophils and lymphocytes in the context of AT1 inhibition following experimental TBI. For this purpose, we employed wild type mice, both with or without neutrophil depletion, as well as recombination activating gene 1—deficient mice (RAG1^−/−^), which are devoid of mature B- and T-lymphocytes. We selected the observation times for each study based on the peak infiltration of these two immune cell types into the brain tissue. Previous research has demonstrated that neutrophils infiltrate the cortical brain tissue in the early stages (between 4 and 72 h), with parenchymal infiltration peaking at 1 day after TBI^18,19^. Therefore, in study A, we utilized observation periods of 4 and 24 h following the TBI. Investigations have shown that lymphocytes begin to invade the cerebral tissue from the third day following the injury^6,18^. Consequently, in study B, we selected the observation periods of 1 and 5 days after TBI. To ensure comparability of the effect of candesartan on both neutropenic and lymphopenic mice, we opted for a 72-h observation period after the TBI for studies C and D, respectively^18^. Our primary endpoints were secondary brain damage, cerebral inflammation, and neurological outcomes.

## Materials and methods

### Animals

A total of 152 male C57BL/6 and RAG1 deficient mice were studied in four different cohorts. In studies A and C, adult male, 8 weeks old, C57BL/6 mice (wild-type (WT) mice; Charles River Deutschland GmbH; Sulzfeld, Germany) were examined. In Study B and D, we investigated male knockout mice lacking RAG1 (RAG1^−/−^; *Translational Animal Research Center* of the University Medical Center of the Johannes Gutenberg-University). Mice were randomly assigned to experimental groups (www.pubmed.de/tools/zufallsgenerator). Experiments and analyses were performed by investigators blind towards group allocation and treatment. The studies were performed with the approval of the Animal Care and Ethics Committee of Rhineland-Palatinate, Germany in accordance with the institutional guidelines of the Johannes Gutenberg University, Mainz (protocol number: 23177-07/G13-1-046) and in compliance with the ARRIVE guidelines. The animals were kept under controlled light and environmental conditions (12-h dark/light cycle, 23 ± 1 °C, 55% ± 5% relative humidity), and had free access to food (Altromin, Germany) and water at all times before and after the experiments.

### Experimental TBI and anesthesia

Animals were anesthetized by intraperitoneal (i.p.) application of midazolam (Hameln pharmaceuticals GmbH; Hameln, Germany), fentanyl (CuraMed, Karlsruhe, Germany) and medetomidine (Dorbene vet; Wien, Austria). An air mixture (40% O_2_ and 60% N_2_) was supplied via facemask in spontaneously breathing mice^20^. Depth of anesthesia was verified by respiration rate and pedal withdrawal reflexes. Rectal temperature was maintained constant at 37 °C by feedback-controlled heating pad (Hugo Sachs, Germany). TBI was performed by controlled cortical impact (CCI) as previously described in detail^8,21^. Briefly, the animal’s head was fixed in a stereotactic frame (Kopf Instruments, USA) and a large craniotomy (4 × 4 mm) was drilled above the right parietal cortex between sagittal, lambdoid, coronal sutures and insertion of the temporal muscle. A custom-fabricated controlled pneumatic impactor (L. Kopacz, Mainz, Germany) was placed perpendicularly to the brain surface and the impactor tip (diameter: 3 mm) centered in the middle of the craniotomy. The impact parameters were as follows: velocity, 8 m/s; duration, 150 ms; brain penetration, 1 mm. Immediately after CCI, the craniotomy was closed with conventional tissue glue (Histoacryl, Braun-Melsungen, Germany) and filament sutures. After the procedure animals were placed in their individual cages and allowed to recover for 6 h in an incubator heated to 33 °C, at a humidity of 35% (IC8000, Draeger, Lübeck, Germany).

### Treatment

#### Application of antibodies for neutrophil granulocyte depletion and control antibodies

For the depletion of neutrophils in WT mice (studies A and C) the Ly6G-specific antibody (anti-mouse, clone 1A8) was used. In the control antibody group, we used the isotype control antibody immunoglobulin IgG2a (rat, clone: 2A3). Both antibodies, anti-Ly6G (1A8) and IgG2a (2A3) (BXCell; West Lebanon, USA) were diluted in PBS with a final concentration of 2.5 mg/mL. We injected 0.2 mL (0.5 mg) of anti-Ly6G antibody (ND) and the same volume of the control IgG2a antibody (Ctrl) intraperitoneally (i.p.) 24 h before (**studies A and C**) and 24 h after experimental TBI (**study C**).

#### Application of the AT1 inhibitor candesartan or vehicle solution

In both studies (**C**, **D**) candesartan, a specific AT1 inhibitor was applied. Candesartan (CV-11974; Tocris bioscience; Bristol, UK) and the vehicle solution were prepared and applied as previously described^8^: The crystalline form of the active drug candesartan was dissolved prior each set of experiments in 0.037 M Na_2_CO_3_ (vehicle solution) in a concentration of 10 µg/mL. The animals received 0.1 mg/kg candesartan (Cand) or vehicle solution (Veh) by subcutaneous (s.c.) injection 30 min after experimental TBI, followed by a daily injection, 24 and 48 h after TBI.

### Experimental protocols (Fig. 1)

**Figure 1:**
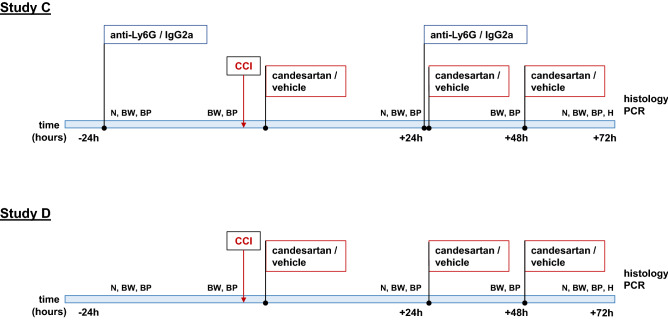
Experimental Timeline. Study C: Effect of AT1 inhibition in neutrophil depleted mice 3 days after TBI: C57BL/6 mice were randomized to treatment (24 h before and 24 h after TBI) with either anti-Ly6G or IgG2a control antibody. They were subjected to controlled cortical impact injury (CCI) and then randomly assigned to additional treatment with candesartan or vehicle solution, performed 30 min after TBI and then repeated daily, 24 and 48 h after TBI. Animals were randomly allocated to four treatment groups: (n = 12/group). To enhance the comparability of the effects on brain tissue infiltration dynamics between neutropenic and lymphopenic mice, we selected an observation time of 72 h after TBI. 72 h after CCI, brains were removed for quantification of lesion volume, cytokine expression and activated microglia (histology, PCR). Study D: Effect of AT1 inhibition in lymphopenic RAG1-deficient mice 3 days after TBI: RAG1-deficient mice (RAG1^−/−^) were randomly assigned to candesartan or vehicle solution treatment (n = 12/group) at 30 min, 24 and 48 h after CCI. 72 h after CCI, lesion volume, cytokine expression and activated microglia were quantified (histology, PCR). CCI, controlled cortical impact; N, neurological assessment; BW, body weight; BP, blood pressure; H, hematology (blood cell count); histology, lesion volume, activated microglia/macrophages; PCR, normalized (PPIA) gene expression (mRNA) of MPO, TNFα, TGFβ, IL1β, IL6 and iNOS.

#### Study A: effect of neutrophil granulocyte depletion 4 and 24 h after TBI

WT mice were randomized to i.p. injection with specific anti-Ly6G (1A8) for neutrophil depletion (ND) or control antibody (Ctrl; IgG2a (2A3)) 24 h before TBI. Lesion volume and cerebral inflammation were determined 4 h (ND-4h, Ctrl-4h; n = 6/group) and 24 h after TBI (ND-24h, Ctrl-24h; n = 8/group). Tissue neutrophil infiltration was quantified by parenchymal myeloperoxidase (MPO) gene expression at both time points. Additionally, neurological outcome was assessed 24 h after TBI. In mice without TBI, neutrophil depletion was controlled by differential blood cell count 24 h after application (ND-0, Ctrl-0; n = 2/group).

#### Study B: effect of RAG1 deficiency mediated lymphopenia 24 h and 5 days after TBI

In RAG1-deficient mice (RAG1^−/−^) and their RAG1^+/+^ wild type litter mates lesion volume and neurology were assessed 24 h (n = 10/group) and 5 days (n = 8/group) after CCI.

#### Study C: effect of AT1 inhibition in neutrophil depleted mice 3 days after TBI

Mice were randomized to treatment (24 h before and repeated 24 h after TBI) with either anti-Ly6G (ND) or IgG2a control antibody (Ctrl). They were subjected to CCI and then randomly assigned to additional treatment with candesartan (Cand) or vehicle solution (Veh), performed 30 min after TBI and then repeated daily, 24 and 48 h after TBI (Fig. 1). Therefore, the animals were randomly allocated to four treatment groups: Ctrl-Cand, Ctrl-Veh, ND-Cand and ND-Veh (n = 12/group). After the 72-h observation period, brains were removed for quantification of lesion volume, cytokine expression and activated microglia. Blood samples were withdrawn for hematological quantification of white blood cells (WBC), lymphocytes and neutrophils. For comparison we used naïve (non-operated) WT mice (n = 6; Fig. 1).

#### Study D: effect of AT1 inhibition in lymphopenic RAG1-deficient mice 3 days after TBI

RAG1-deficient mice were randomly assigned to candesartan or vehicle solution treatment (RAG1^−/−^-Cand, RAG1^−/−^-Veh; n = 12/group) at 30 min, 24 and 48 h after TBI (Fig. 1). As in study C, 72 h after TBI, lesion volume, cytokine expression and activated microglia were quantified and hematologic assessment was performed. Additionally, we used naïve RAG1^−/−^ mice (n = 6; Fig. 1).

### Measurement of physiological parameters

Before, and after experimental TBI body weight of each mouse was controlled. Blood pressure was measured 5 min before and after CCI under general anesthesia at the tail using a modified NIBP system (RTBP 2000, Kent Scientific, Torrington, USA; A/D converter: PCI 9112, Adlink Technology, Taiwan; software: Dasylab 5.0, measX, Germany; Flexpro 6.0, Weisang, Germany) as previously described^20^. Additionally, blood pressure values were determined in awake animals daily for 8 days before (training phase) and for 2 days after CCI. Perioperative body temperature was measured by a rectal temperature probe (Physitemp; Clifton, NJ, USA).

### Assessment of functional outcome

In **studies A, C and D** neurological outcome was assessed using the rotarod performance test (Heidolph Instruments GmbH &Co.; Schwabach, Germany) as previously described^22–25^. After a pre-training phase (mice remained on a rotating rod for 20 s at 4 rpm) two days before TBI, the time to fall from the accelerating rod in the 2-min test period was registered. This test assesses coordination and motoric function and was performed 1 day before, 24 and 72 h after CCI. In **study B** functional outcome was determined by Neurological Severity Score^26^. In addition to the rotarod test, in **studies C and D**, functional outcome was also determined by modified neurological severity score (mNSS; modified after Tsenter et al.^26^) 1 day before and 24 and 72 h after CCI^4^. To calculate mNSS, general behavior, alertness, motor ability and balance were rated with 6 different tasks. Each task was scored from 0 (normal) up to 3 (failed task). The mNSS ranges from 0 (healthy) to 16 (severely impaired) points^27^ (Table 1). All neurological tests were performed by investigators blinded towards experimental group allocations.

**Table 1:** Modified neurological severity score (mNSS).

		Points
1. Exit from circle	< 30 s	0
	For 30 s	1
	For 60 s	2
	> 2 min	3
2. Startle reflex	Present	0
	Absent	1
3. General behavioral deficit		
Seeking behavior	Present	0
	Absent	1
Walk straight	Present	0
	Absent	1
4. Coordination (Criteria: 0P: no impairment; 1P: feet misplacement, unstable; 2P: stops moving)		
Beam walking 3 cm	Score	(0–2)
Beam walking 1,5 cm	Score	(0–2)
Beam walking 1 cm	Score	(0–2)
5. Balance (Criteria: 0P: grips stick with 4 paws; 1P: 1–4 paws not gripping)		
Round stick	Score	(0–1)
Square stick	Score	(0–1)
6. Motor deficit		
Hemiparesis	Absent	0
	One Foot	1
	Present hemiparesis	2

The modified Neurological Severity Score (mNSS) was designed on the basis of the Neurological Severity Score introduced by Tsenter et al.^26^. The mNSS focusses on motoric function and behavioral deficits and was performed 1 day before CCI and on posttraumatic day 1 and 3 (day 5 in study C) after experimental TBI.

### Flow cytometry and blood cell count

**Figure 2:**
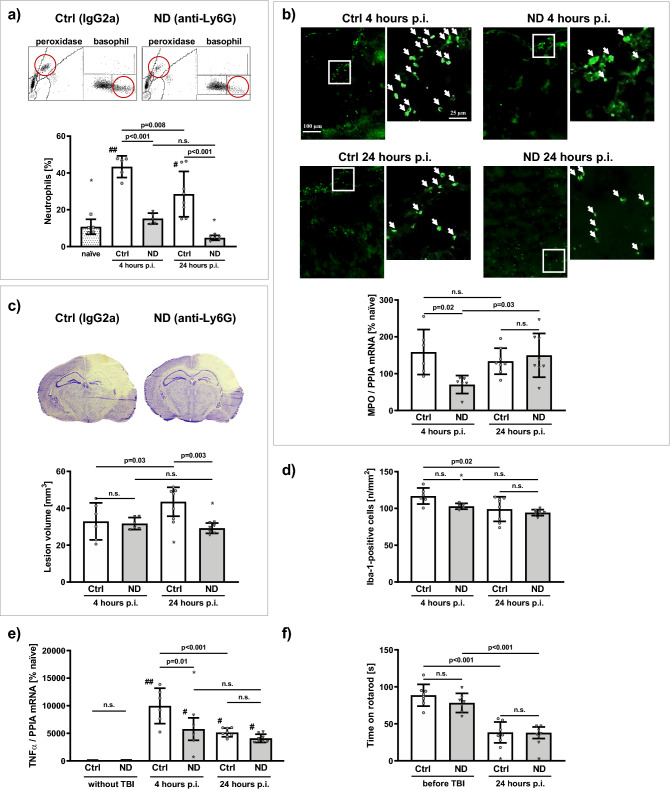
Depletion of neutrophils reduces brain damage after TBI (Study A). Male C57BL/6 mice were randomized to i.p. injection with specific anti-Ly6G (1A8) for neutrophil depletion (ND) or control antibody (Ctrl; IgG2a (2A3)) 24 h before TBI. With respect to the maximum brain tissue infiltration of neutrophils, lesion volume and cerebral inflammation were determined 4 h (ND-4h, Ctrl-4h; n = 6/group) and 24 h after TBI (ND-24h, Ctrl-24h; n = 8/group; p.i. = post injury). In diagrams control antibody IgG2a treated groups are depicted in white (Ctrl), neutrophil granulocyte depleted, anti-Ly6G treated groups are shown in light grey (ND). (**a**) Neutrophils in the WBC count: (**a**) top: representative cytograms of mice treated with control antibody IgG2a (Ctrl), and of mice treated with anti-Ly6G (ND) for neutrophil depletion. The cytograms, obtained with the ADVIA 2120i Hematology System contain the two channels of accurate quantification: peroxidase and basophil (lobularity/nuclear density). The neutrophils are marked by the red circle. (**a**) Bottom: neutrophil granulocyte count [% WBC]: Within the first 24 h after TBI, at both time points, there was a significant increase of neutrophils in Ctrl mice, compared to naïve (##p < 0.001; #p < 0.05), and compared to ND mice (p < 0.001). Neutrophil depletion with anti-Ly6G significantly reduced neutrophils at 4 and 24 h after TBI (p.i.). In Ctrl mice, after the neutrophil peak at 4 h, neutrophil fraction decreased by 33% at 24 h (p.i.). (**b**) Assessment of neutrophil infiltration: (**b**) top: representative anti-Gr1 immunohistochemistry images of tissue infiltration of neutrophils (marked by white arrows; inside the lesion, normal and zoomed image): 4 h after TBI neutrophil infiltration is reduced by ND, compared to Ctrl. At 24 h after TBI, however tissue neutrophil infiltration was not different between ND and Ctrl. (**b**) Bottom: quantitative assessment of the neutrophil infiltration was performed by the gene expression analysis of the neutrophil marker MPO within the ipsilateral hemisphere (mRNA expression, normalized with PPIA, expressed as % naïve, normalized expression: 5.48 ± 0.17 × 10^–6^ mRNA/PPIA). The administration of the anti-Ly6G neutrophil-depleting antibody 24 h before CCI resulted in a reduced gene expression of MPO in the brain tissue 4 h after CCI, that is 28 h after antibody administration. After 24 h, however, MPO gene expression in ND increased significantly to Ctrl-levels, there was no difference between ND and Ctrl. (**c**) Lesion volume assessed 4 h and 24 h after CCI in Nissl-stained sections: (**c**) top: typical, representative pictures of Nissl-stained coronal sections at bregma level − 1.94 mm in mice treated with Ctrl (IgG2a) compared to ND mice (anti-Ly6G) at 24 h after CCI. (**c**) bottom: Neutrophil depletion reduced lesion volume [mm^3^] 24 h after TBI by 33%. (**d**) Number of activated microglia was assessed in cortical perilesional tissue in a ROI (0.52 × 0.65 mm^2^) adjacent to the injury (bregma − 1.28 mm), expressed as number of Iba-1 positive cells/mm^2^. (**e**) Gene expression of TNFα was assessed by real-time qPCR, normalized with PPIA, and expressed as % naïve (normalized expression: 2.3488 ± 1.3114 × 10^–6^ mRNA copies/PPIA). In mice without trauma (ND-0, Ctrl-0; n = 2/group) there was no difference between Ctrl and ND. At 4 h after TBI Ctrl TNFα expression was significantly higher than ND (^#^p < 0.05 and ^##^p < 0.001 vs. without TBI). (**f**) Neurological outcome was assessed before and 24 h after TBI by time spent on the rotarod [s]. (**a**–**e**) Ordinary one-way analysis of variance (ANOVA) with Holm-Šidák’s multiple comparisons test. (**f**) Mixed effects analysis (REML) with Holm-Šidák’s multiple comparisons test; p < 0.05; n.s. = not significant. Data is presented as mean ± SD. Excluded outlier values are presented with an asterisk (*).

At the end of observation period, great care was taken to perform accurate routine differential blood cell count. In deep anesthesia, EDTA anti-coagulated blood samples were taken from the retro-orbital veins as previously described^28^. The differential blood cell count was obtained via the ADVIA 2120i Hematology system by a medical technician specialized in murine blood analyses and blinded to experimental group allocation. The ADVIA 2120i is a Ly6G-independent full automated veterinary flow cytometry analyzer, validated for murine blood analyses. The analyses were performed after the standardized protocol of the Institute of Clinical Chemistry and Laboratory Medicine of the University Medical Center of Mainz (ADVIA 2120i Hematology System; Siemens Healthcare, Erlangen, Germany; https://www.siemens-healthineers.com/en-us/hematology/systems/advia-2120-hematology-system-with-autoslide). The hematology analyzer ADVIA 2120i is a flow cytometry-based system that uses laser light scatter to differentiate and count WBC in two different ways: the peroxidase method and the lobularity/nuclear density method^29^. The peroxidase method uses the myeloperoxidase (MPO) to detect, differentiate and quantify the WBC when they pass through the flow cell. With the help of an optical system all WBC are counted, and peroxidase reagents are used to distinguish between MPO-positive cells, such as neutrophils, eosinophils, and monocytes, and peroxidase-negative cells, which include lymphocytes, and basophils^29^. The cells absorb light in proportion to the amount of peroxidase stain present, and this peroxidase activity parameter is represented on the x-axis of the peroxidase cytogram (Fig. 2a). Cells scatter light in proportion to their size, and this cell size parameter is represented on the y-axis of the cytogram (Fig. 2a). When the light-scatter and absorption data are plotted, distinct populations or clusters are formed, and cluster analysis is applied to identify different cell populations^29^. In the lobularity/nuclear density channel, surfactant and phthalic acid are used to lyse red blood cells and platelets, and to strip away the cytoplasmic membrane from all leukocytes, except basophils. Cells are then counted, and classified, according to size, lobularity, and nuclear density^29^. By the cluster analysis, the polymorphonuclear cells (neutrophils), the mononuclear leukocytes as well as the basophils are quantified.

### Histologic and immunohistochemical evaluation

According to our previous protocol^4^ brains were removed in deep anesthesia. For tissue evaluation, the brains were frozen in powdered dry ice and stored at − 20 °C. They were then cut in coronal plane with a cryostat (HM 560 Cryo-Star, Thermo Fisher Scientific, Walldorf, Germany) as previously described in detail^8^. The first slide was defined according to the first section corresponding to bregma + 3.14 mm in the Mouse Brain Library (www.mbl.org). 16 sections (12 and 20 µm) were collected at 500 µm-intervals, placed on Superfrost + TM slides (Thermo Fisher Scientific, Germany). In cresyl violet (Merck, Darmstadt, Germany) stained sections (12 µm), the total area of both hemispheres and the injured brain tissue area were determined for each section and animal using a computerized image analysis system (Delta Pix Insight; Maalov, Denmark) by an investigator blind to the group allocation. The total hemispheric brain volumes and the lesion volumes were calculated by following formula: 0.5 [mm] × (area of slide 1 [mm^2^] + area of slide 2 [mm^2^] + … + area of slide 16 [mm^2^])^4,8^. Immunohistochemical staining was performed as described before^27^. Briefly, cryosections (20 µm) were fixed in 4% paraformaldehyde in phosphate buffered saline (PBS), incubated with blocking solution (5% goat serum, 1% bovine serum albumin, and 0.1% TX-100 in PBS) for 1 h at room temperature. Primary antibodies specific for anti-ionized calcium-binding adapter molecule-1 (Iba-1; rabbit anti-mouse, anti-Iba-1 antibody; Wako Chemicals GmbH, Neuss, Germany), or Gr1 (rat anti-Ly6g + Ly6c, clone RB6-8C5, Abcam, UK) were applied in blocking solution overnight at 4 °C. The sections were washed, incubated with secondary biotin-conjugated antibodies (goat anti-rabbit IgG; Merck; Darmstadt, Germany) and processed according to the manufacturer’s instructions using Vectastain Elite ABC Kit (Vector Laboratories, Burlingame, USA), or fluorophore conjugated secondary antibodies (goat anti-rabbit IgG, Biotinylated; Merck; Darmstadt, Germany or goat anti-rat IgG Alexa Fluor 488, Thermo Fisher). Images of anti-Iba-1 immunostaining were taken at × 20 magnification (Axiovert, Zeiss, Germany), for anti-Gr-1 immunostaining at × 10 magnification (Keyence, BZ-X800). The total number of Iba-1-positive cells were counted at bregma − 1.28 mm in a region of interest (ROI) of 0.52 × 0.65 mm^2^ in the cortical tissue adjacent to the lesion by an investigator blind to randomization, using ImageJ software (National Institutes of Health, USA). Iba-1-immunolabeled cells with appropriate morphology and appearance^30^ were identified as activated microglia/macrophages and assessed in the ROI (0.52 × 0.65 mm^2^ in the cortical tissue adjacent to the lesion). The rationale for counting Iba-1-positive cells in an area adjacent to the lesion rather than within the lesioned area was, that inside the lesion, where the tissue is essentially destroyed, microglia/macrophages are almost absent. In the perilesional area, however, there is a robust activation of microglia/macrophages. Results are presented in number of activated Iba-1 positive cells/mm^2^. Unfortunately, technical difficulties prevented us from obtaining an adequate quantitative assessment of Gr1-positive cells. Therefore, we present qualitative images with Gr1 staining (Fig. 2b).

### Gene expression analysis

Brain tissue samples from the lesion and perilesional area of 500 µm coronal cryostat sections between histologic slice intervals were collected, snap frozen in liquid nitrogen, stored at − 80 °C. As described previously in detail^4,31^, after tissue sampling, extraction of mRNA and cDNA synthesis qPCR were performed (lysis: Qiazol-reagent, Qiagen, Hilden, Germany; homogenization: MM300 mill mixer, Retsch, Haan, Germany; RNA isolation: RNeasy Lipid Tissue Kit, Qiagen, Hilden, Germany; RNA concentration determined by spectrometer: NanoVue System, GE Healthcare Europe, Munich, Germany; RNA to cDNA reverse transcription Verso cDNA Kit, ABgene, Hamburg, Germany; cDNA amplification: real-time Ligthcycler 480 PCR System, Roche). PCR fragments of all applied genes were generated by PCR on an Eppendorf Thermocycler gradient (Eppendorf, Hamburg, Germany). The PCR products were purified with QIA quick PCR Purification Kit (Qiagen) according to the manufacturer´s instructions, and the DNA concentration was determined using NanoVue. A standard curve for absolute quantification was generated with PCR DNA for each PCR product (10^1^–10^7^ DNA copies/µl), showing similar and good efficiency (90–110%; LightCycler Software, Roche) and linearity. Equal amounts of cDNA (1 µL) of each sample were analyzed in duplicates and amplified by real-time Lightcycler 480 PCR System (Roche). Real-time RT PCR kits were used according to the manufacturer´s instructions. All assays were conducted by an investigator blinded to group allocation. Using mouse-specific primers and probes (Table 2) and optimized temperature conditions for qPCR, absolute copy numbers of the target genes, tumor necrosis factor α (TNFα), transforming growth factor β (TGFβ), interleukin 1β (IL1β), interleukin 6 (IL6), inducible nitric oxide synthase (iNOS) and myeloperoxidase (MPO) were calculated, and were then normalized against the absolute copy numbers of cyclophilin A (PPIA)^4,6,8,32^. The reference gene PPIA was chosen as single normalizer^33^ based on recent findings in our housekeeping gene study^32^. In order to improve comparability of the mRNA expression data between different treatment groups, and to eliminate qPCR kit dependent differences and limitations, qPCR data was normalized with PPIA and then related to normalized naïve target gene expression from naïve tissue samples from the corresponding brain region^34^. Therefore, normalized target gene expression values are expressed as % naïve expression^4^.

**Table 2:** Primers for the quantitative real-time PCR.

PCR assay (amplicon size)	Oligonucleotide sequence (5′–3′)	GenBank no.
PPIA (146 bp)	F: 5′-GCGTCTSCTTCGAGCTGTT-3′	NM_008907
	R: 5′-RAAGTCACCACCCTGGCA-3′	
IL1β (348 bp)	F: 5′-GTGCTGTCGGACCCATATGAG-3′	NM_008361
	R: 5′-CAGGAAGACAGGCTTGTGCTC-3′	
IL6 (471 bp)	F: 5′-TCGTGGAAATGAGAAAAGAGTTG-3′	NM_031168
	R: 5′-TATGCTTAGGCATAACGCACTAG-3′	
TNFα (212 bp)	F: 5′-TCTCATCAGTTCTATGGCCC-3′	NM_013693
	R: 5′-GGGAGTAGACAAGGTACAAC-3′	
TGFβ (142 bp)	F: 5′-CTTCAATACGTCAGACATTCGGG-3′	NM_011577
	R: 5′-GTAACGCCAGGAATTGTTGCTA-3′	
iNOS (312 bp)	F: 5′-TGTGTCAGCCCTCAGAGTAC-3′	NM_010927
	R: 5′-CACTGACACTYCGCACAA-3′	
MPO (220 bp)	F: 5′-ACACCCTCATCCAACCCTTC-3′	NM_010824.2
	R: 5′-TGCTCAAATAGTCGCTCCC-3′	

Mouse specific primers and probes for quantitative real-time polymerase chain reaction (qPCR): cyclophilin A (PPIA), interleukin 1β (IL1β), interleukin 6 (IL6), tumor necrosis factor α (TNFα), transforming growth factor β (TGFβ), inducible nitric oxide synthase (iNOS), and myeloperoxidase (MPO); F: forward, R: reverse.

### Statistical analysis

All experiments were randomized and performed by investigators blinded toward the treatment groups (computer-based randomization: www.pubmed.de/tools/zufallsgenerator). In order to determine the required sample size, the a priori power analysis using G ∗ Power^35^ was performed with the main variable, the primary endpoint, lesion volume data from previously published studies^4,8^. Therefore, based upon the data of these studies the present a priori power analysis was performed to determine an effect size of d 1.75, with an actual standard statistical power (1−β) of 0.95, and a significance level (α) of 0.05 and a sample size per group of n = 7. In order to have a sufficient power, we decided to have larger sample sizes (n = 8–12, per group)^36^. Statistical analysis was performed using the GraphPad Prism 8 Statistical Software (GraphPad Software Inc., La Jolla, CA, USA). Data distribution was tested by Shapiro-Wilks test. The comparisons of parametric and non-parametric data between two independent groups were done using the Welch-t test and the Wilcoxon rank sum test, respectively. For the statistical analysis of mNSS we performed ANOVA on ranks with the Kruskal–Wallis test, corrected for multiple comparisons using the Dunn’s test. In this multi-arm parallel group randomized trial, for comparison of multiple independent groups, if the Shapiro–Wilk normality test was passed, one-way analysis of variance (one- way ANOVA) with post-hoc Holm-Šidák comparisons test (comparisons between all groups) was employed. In experimental groups where two separate treatment factors (neutrophil depletion and AT1 inhibition) are present, a two-way analysis of variance (two-way ANOVA) was performed. Physiologic data, blood cell count, lesion volumes, number of activated microglia and mRNA expression data were compared between experimental groups with two-way ANOVA and post hoc with all-pairwise multiple comparison procedures (Holm-Šidák method). To evaluate group differences in repeated measurements from the same animals (body weight, systolic blood pressure), repeated measures (RM) two-way ANOVA (two-factor repetition) was applied (factors: treatment and time), followed by Šidák’s multiple comparisons test. Whenever there were missing values in the repeated measures dataset and a two-way ANOVA was not possible, repeated measures (mNSS, rotarod) data were analyzed with the mixed effect model using the restricted maximum likelihood (REML) method with Holm Šidák’s multiple comparison test. The p values were adjusted for multiple comparisons. Values of p < 0.05 were considered significant. To identify outliers in our dataset, we employed a combination of the iterative Grubb's test and Z-scores using the 2 standard deviations method with a confidence interval of 95%, where appropriate. Additionally, we have included scatter plots in the figures to depict the data distribution effectively. Data are presented as mean and standard deviation (mean ± SD).

### Ethics approval

The studies were performed with the approval of the Animal Care and Ethics Committee of Rhineland-Palatinate, Germany in accordance with the institutional guidelines of the Johannes Gutenberg University, Mainz (protocol number: 23177-07/G13-1-046).

## Results

### Perioperative physiological parameters were stable in all groups

Peri- and intraoperative body temperature and systolic blood pressure were in all mice within physiological range, without considerable difference between groups (Table 3). As published earlier, in our standardized anesthesia and operation setting, values were stable and within physiological limits^6^.

**Table 3:** Systolic blood pressure after TBI.

Treatment groups	0 (CCI)	6 h	24 h	36 h
Ctrl—Vehicle	*119* ± *17*	112 ± 15	123 ± 12	114 ± 13
Ctrl—Candesartan	*111* ± *14*	106 ± 22	120 ± 14	112 ± 19
ND—Vehicle	*101* ± *17*	104 ± 17	128 ± 22	122 ± 18^§^
ND—Candesartan	*112* ± *17*	102 ± 15	145 ± 5^#,$^	133 ± 21^$^
RAG1^−/−^—Vehicle	*113* ± *21*	112 ± 13	136 ± 16	112 ± 22
RAG1^−/−^—Candesartan	*110* ± *11*	112 ± 15	119 ± 16	117 ± 23

Systolic blood pressure [mmHg] during 36 posttraumatic hours was within physiologic range and not affected by low dose candesartan treatment. Time point 0 represents intraoperative measurement immediately after CCI induction under general anesthesia (in italic characters). At the following time points, measurements were performed in awake animals (normal characters). At certain time points there were significant differences, intergroup (^#^p < 0.05 ND-Cand vs. Ctrl-Cand) and within the groups (ND-Veh: ^§^p < 0.05 vs. 0 (CCI); ND-Cand: ^$^p < 0.05 vs. 6 h).

### Low-dose candesartan treatment did not influence blood pressure after CCI

As the specific AT1 antagonist candesartan is used for the treatment of arterial hypertension, we determined its influence on arterial blood pressure. In the present study, low dose (0.1 mg/kg) candesartan treatment did not to alter blood pressure, as shown in previous studies^4,8^. During the observation period, in all groups, blood pressure was within physiological range (Table 3).

### After TBI bodyweight was not affected by anti-Ly6G, RAG1-deficiency or AT1 inhibition

In naïve mice bodyweight was 24.7 ± 2.2 g. Before TBI, initial bodyweight was in Ctrl-4h mice 25.6 ± 0.9 and in ND-4h mice 25.5 ± 1.1 g. After 24 h, bodyweight was reduced in both Ctrl and ND, without any difference (before: 25.8 ± 0.7 and 26.0 ± 1.2 g; 24 h after TBI: 24.1 ± 1.2* and 24.3 ± 1.3*, for Ctrl and ND, respectively, *p < 0.001 vs. before TBI). The body weight of RAG1^+/+^ and RAG1^−/−^ were similarly reduced after CCI (24-h investigation: before: 22.7 ± 0.8 and 24.2 ± 1.9 g; 24 h after TBI: 20.3 ± 1.2* and 21.5 ± 1.7* g, for RAG1^+/+^ and RAG1^−/−^, respectively, *p < 0.001 vs. before TBI). In the 5-days investigation, after a body weight loss at 1 day after CCI, RAG1^+/+^ and RAG1^−/−^ animals regained weight at 5 days after CCI in both groups (before TBI: 22.4 ± 1.1 and 22.2 ± 1.2 g, 1 day after TBI: 20.6 ± 1.2*^#^ and 20.4 ± 0.5*^#^ and 5 days after TBI: 22.3 ± 0.9 and 21.8 ± 1.5, for RAG1^+/+^ and RAG1^−/−^, respectively, *p < 0.001 vs. before and ^**#**^p < 0.001 1 vs. 5 days after TBI). In both investigations RAG1 deficiency did not affect posttraumatic body weight loss. In all groups, there is a significant posttraumatic decrease of bodyweight, with a minimum on day 2 and an increase on day 3. However, neither neutrophil depletion, nor RAG1 deficiency, nor AT1 inhibition affected posttraumatic body weight loss, compared to control antibody, wild type or vehicle solution treated mice (Table 4).

**Table 4:** Pre- and postoperative body weight development.

Treatment groups	Bodyweight [g] before TBI	Bodyweight [g] 24 h after TBI	Bodyweight [g] 48 h after TBI	Bodyweight [g] 72 h after TBI
Ctrl-Veh	24.9 ± 1.3	23.4 ± 1.6*	23.4 ± 1.2*	24.2 ± 1.1*^,$,#^
Ctrl-Cand	24.5 ± 2.0	23.1 ± 2.1*	23.1 ± 2.0*	23.9 ± 2.1*^,$,#^
ND-Veh	24.8 ± 1.9	23.3 ± 1.7*	23.3 ± 1.4*	24.1 ± 1.5*^,$,#^
ND-Cand	24.8 ± 0.8	23.1 ± 0.8*	22.9 ± 0.8*	23.9 ± 0.8*^,$,#^
RAG1^−/−^-Veh	24.0 ± 2.3	23.6 ± 2.3*	22.9 ± 2.1*^,$^	23.5 ± 2.2*^,#^
RAG1^−/−^-Cand	23.9 ± 2.9	23.3 ± 3.0*	22.4 ± 2.8*^,$^	23.3 ± 2.7*^,#^

Bodyweight was assessed before induction of anesthesia and experimental TBI and 24, 48 and 72 h after TBI (*p < 0.05 vs. before TBI; ^$^p < 0.05 vs. 24 h after TBI; ^#^p < 0.05 vs. 48 h after TBI).

### Neutropenia was achieved with anti-Ly6G in WT, lymphopenia was present in RAG1^−/−^ mice

Differential blood cell counts were performed (Table 5). In animals without TBI, neutrophils were reduced from 10.8 ± 4.1% (0.64 ± 0.25 × 10^3^ cells/µL) in naïve WT mice to 2.9 ± 0.4% (0.15 ± 0.01 × 10^3^ cells/µL; ND-0) in anti-Ly6G, compared to control antibody treated mice (IgG2A): 10.1 ± 1.3% (0.61 ± 0.30 × 10^3^ cells/µL; Ctrl-0). In Ctrl-mice WBC were reduced 4 h after TBI by 40% (Ctrl-4h). However, 24 h (Ctrl-24h) and 72 h after TBI (Ctrl-Veh and Ctrl-Cand) WBC values corresponded to initial naïve values. In ND-mice WBC reduction was more distinct and sustaining (ND-4h and-24h), with lasting relatively low WBC count to day 3 after TBI (ND-Veh and ND-Cand). In non-neutrophil-depleted mice, as a response to TBI, there was a shift from lymphocyte-dominated (84%) WBC to an elevation of the neutrophil fraction from 11% (naïve), to 43%, followed by a continuous decrease by 33% to 29 at 24 h (p < 0.05; Fig. 2a) and to 16% at 72 h in Ctrl-treated mice after TBI. In ND mice, in contrast, after TBI there is no initial elevation of neutrophils. Moreover, 24 and 72 h after TBI there is a significant neutropenia in ND-mice alongside elevated lymphocyte counts. Candesartan did not affect neutrophil counts (Table 5). Naïve RAG1^−/−^ mice are leukopenic, reduced lymphocyte counts (40%) are compensated by elevated neutrophil numbers (34%). After TBI in RAG1^−/−^ mice there is a decrease of WBC in vehicle treated mice, whereas in candesartan treated mice the decrease is not significant. In RAG1^−/−^ there is also a decrease of lymphocytes (25%) and an increase of neutrophils to 50%, after TBI, that are not affected by AT1 inhibition (Table 5). There is a sustaining posttraumatic decrease of monocytes in all groups. In candesartan treated ND mice, however there is a normalization of monocyte count 3 days after TBI. At 4 h after TBI there is a transient elevation of hemoglobin and hematocrit, with normalization of these parameters at 24 and 72 h after TBI. Platelets were within a physiological range in all groups at all time points and not affected by the treatment (Table 5).

**Table 5:** Blood cell count.

Study A					
	naïve	Ctrl-4h	ND-4h	Ctrl-24h	ND-24h
White blood cells (× 10^3^ n/µL)	5.7 ± 0.6	3.4 ± 1.0*	2.2 ± 0.4**	4.6 ± 1.6	3.5 ± 0.9*
Neutrophils (%)	10.8 ± 4.1	43.4 ± 5.9**	15.2 ± 3.0^##^	28.5 ± 12.3*^,§^	4.8 ± 1.3^##^
Lymphocytes (%)	83.6 ± 3.6	52.4 ± 6.0**	80.1 ± 2.6^##^	65.6 ± 11.1**^,§^	90.2 ± 2.1^##^
Monocytes (%)	1.0 ± 0.2	0.5 ± 0.2*	0.4 ± 0.2**	0.7 ± 0.2*	0.5 ± 0.1**
Hemoglobin (g/dL)	14.7 ± 1.2	16.2 ± 0.9	17.3 ± 0.4**	15.2 ± 0.8	14.8 ± 0.6^§§^
Platelets (× 10^3^ n/µL)	1277 ± 125	1130 ± 117	1000 ± 70	1115 ± 220	1087 ± 125
* p < 0.05 and ** p < 0.001 vs. naïve; ^§^ p < 0.05 and ^§§^ p < 0.001 vs. 4 h; ^##^ p < 0.001 vs. Ctrl					

Study C					
	naïve	Ctrl-Veh	Ctrl-Cand	ND-Veh	ND-Cand
White blood cells (× 10^3^ n/µL)	5.7 ± 0.6	4.2 ± 1.1	4.6 ± 1.3	3.9 ± 0.8*	3.6 ± 1.0*
Neutrophils (%)	10.8 ± 4.1	16.0 ± 3.0*	15.2 ± 2.7*	6.8 ± 2.5^**##**^	5.8 ± 2.7*^,**##**^
Lymphocytes (%)	83.6 ± 3.6	78.5 ± 2.5*	80.3 ± 2.6	88.4 ± 4.5^**##**^	87.3 ± 3.4^**##**^
Monocytes (%)	1.0 ± 0.2	0.4 ± 0.2**	0.5 ± 0.2**	0.5 ± 0.3**	0.7 ± 0.3^**#**,$^
Hemoglobin (g/dL)	14.7 ± 1.2	14.4 ± 0.7	14.2 ± 1.0	15.2 ± 0.8	14.4 ± 0.8
Platelets (× 10^3^ n/µL)	1277 ± 125	1253 ± 99	1377 ± 131	1124 ± 129	1234 ± 191
* p < 0.05 and ** p < 0.001 vs. naïve; ^#^ p < 0.05 and ^##^ p < 0.001 vs. Ctrl., and ^$^ p < 0.05 vs. ND-Veh					

Study D					
	RAG1^−/−^ naïve	RAG1^−/−^-Veh	RAG1^−/−^-Cand		
White blood cells (× 10^3^ n/µL)	1.3 ± 0.6	0.7 ± 0.3*	0.9 ± 0.3		
Neutrophils (%)	33.7 ± 5.2	44.5 ± 8.9*	50.0 ± 6.4**		
Lymphocytes (%)	40.3 ± 5.6	25.3 ± 3.5**	25.7 ± 5.2**		
Monocytes (%)	4.0 ± 0.5	1.4 ± 0.6**	1.3 ± 0.7**		
Hemoglobin (g/dL)	15.1 ± 0.6	14.5 ± 0.5*	14.4 ± 0.4*		
Platelets (× 10^3^ n/µL)	1404 ± 100	1476 ± 79	1499 ± 145		
* p < 0.05 and ** p < 0.001 vs. naïve					

Routine differential blood cell count was performed by a medical technician, blinded to groups allocation, with a validated fully automated veterinary flow cytometry system (ADVIA2120i) in the laboratories of the Institute of Clinical Chemistry and Laboratory Medicine, University Medical Center of Johannes Gutenberg-University Mainz.

### Study A: effect of Neutrophil granulocyte depletion

#### Neutrophil depletion reduced tissue infiltration of neutrophils only at 4 h after TBI

To detect the neutrophil infiltration into injured brain tissue we analyzed the gene expression of MPO, considered a neutrophil marker^37^, by qPCR (normalized naïve expression: 0.00000548 ± 0.00000017 mRNA/PPIA). In the first 4 h after TBI the gene expression of MPO is elevated to 159 ± 60% naïve in Ctrl mice. Neutrophil depletion by anti-Ly6G reduced MPO expression within the injured brain tissue by 56% (70 ± 24% naïve; p < 0.05), compared to Ctrl mice, at 4 h after CCI (Fig. 2b). However, at 24 h after TBI (i.e.: 48 h after application of anti-Ly6G), MPO gene expression of ND mice increased to the level of Ctrl (p < 0.05%; Fig. 2b), despite reduced neutrophils in the WBC count of ND mice (Fig. 2a).

#### Neutrophil granulocyte depletion reduced lesion volume 24 h after TBI

Lesion volume was assessed 4 h and 24 h after CCI in Nissl-stained sections (Fig. 2c**)**. In Ctrl mice, lesion volume increased from 32.9 ± 10.0 mm^3^ (21.0 ± 6.5% contralateral hemisphere volume, %-clh) at 4 h, to 43.6 ± 7.8 mm^3^ (27.7 ± 5.8%-clh) at 24 h after TBI (p < 0.05). In ND mice, there was no increase in lesion volume at 24 h (29.2 ± 2.8 mm^3^; 18.7 ± 1.7%-clh) compared to 4 h after TBI (31.7 ± 3.2 mm^3^; 20.1 ± 2.5%-clh). Moreover, neutrophil depletion reduced the lesion volume 24 h after TBI compared to control group by 33% (p < 0.05, Fig. 2c).

#### Neutrophil granulocyte depletion reduced cerebral inflammation

Number of Iba-1-positive cells was assessed at bregma − 1.28 mm in the cortical perilesional tissue in a ROI (0.52 × 0.65 mm^2^) adjacent to the injury, results are expressed as number of Iba-1-positive cells/mm^2^. Compared to 4 h after TBI (n = 117 ± 11/mm^2^), there was a decrease of Iba-1-positive cells 24 h after TBI in Ctrl mice (n = 99 ± 17/mm^2^; p < 0.05). However, neutrophil depletion did not affect the number of Iba-1-positive microglia/macrophages in the first 24 h after TBI (Fig. 2d). Cytokine gene expressions were determined by qPCR: normalized naïve expressions [mRNA/PPIA]: IL1β: 0.00197 ± 0.00044; IL6: 0.06063 ± 0.01640; TNFα: 2.3488 ± 1.3114 × 10^–6^; TGFβ: 0.00560 ± 0.00078; iNOS: 0.00141 ± 0.00034. Gene expressions of experimental groups are expressed as % naïve expression. In mice without experimental TBI there was no difference in gene expressions between treatment groups (e.g., IL1β: 87 ± 10 and 90 ± 5, % naïve expression for Ctrl-0 and ND-0, respectively). Within the first 24 h after TBI, gene expressions increased in all groups. Though, posttraumatic expressions of IL1β, IL6, TGFβ and iNOS did not differ between treatment groups. Posttraumatic increase of TNFα expression, however, was different. At 4 h after TBI TNFα expression (9969 ± 3211% naïve) was significantly higher in Ctrl than in ND (5771 ± 2024% naïve, p < 0.05; Fig. 2e). However, at 24 h after TBI TNFα expressions were similar in both treatment groups (Ctrl: 5151 ± 783 and ND: 4096 ± 748% naïve, Fig. 2e).

#### Neutrophil granulocyte depletion did not affect neurological outcome

Neurological outcome was assessed before and 24 h after TBI by rotarod performance test. One day after TBI there was a marked neurological impairment in both groups (from 89 ± 15 and 78 ± 12 s before TBI to 38 ± 14 and 38 ± 8 s at 24 h after TBI, for Ctrl and ND, respectively, p < 0.001). However, neutrophil depletion did not alter neurological outcome compared to control group (Fig. 2f).

### Study B: effect of Lymphopenia in RAG1 deficient mice

#### Lesion volume in RAG1^−/−^ mice was reduced 1 and 5 days after TBI compared to RAG1^+/+^

**Figure 3:**
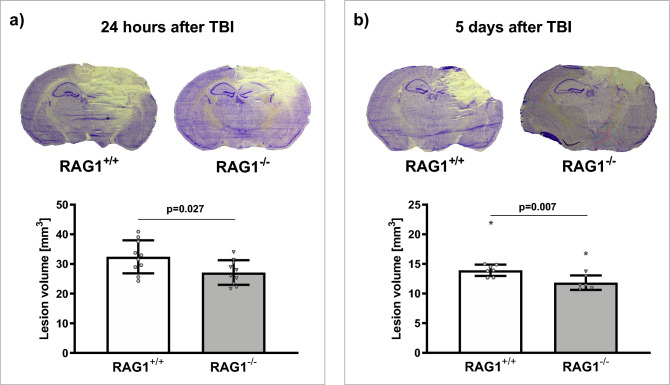
RAG1 deficiency reduced lesion volume compared to wild type mice (Study B). In wild type mice (RAG1^+/+^), shown in white, and their RAG1 deficient litter mates (RAG1^−/−^), depicted in grey, lesion volume was assessed in Nissl-stained sections 24 h (n = 10/group, **a**), and 5 days (n = 8/group, **b**), with respect to lymphocyte brain tissue infiltration time, after CCI. (**a**) Top: typical, representative pictures of Nissl-stained coronal sections at bregma level − 1.94 mm in wild type (RAG1^+/+^) compared to RAG1 deficient mice (RAG1^−/−^) at 24 h after TBI. (**a**) Bottom: in RAG1^−/−^-mice lesion volume was reduced compared to RAG1^+/+^ mice by 17% at 24 h after TBI. (**b**) Top: typical, representative pictures of Nissl-stained coronal sections at bregma level − 1.94 mm in wild type (RAG1^+/+^) compared to RAG1 deficient mice (RAG1^−/−^) at 5 days after TBI. (**b**) Bottom: 5 days after TBI, lesion volume was reduced in RAG1^−/−^ by 16% compared to RAG1^+/+^. Unpaired t-test with Welch’s correction. Data is presented as mean ± SD; p < 0.05. Excluded outlier values are presented with an asterisk (*).

Lesion volume was assessed in RAG1^−/−^ mice and their RAG1^+/+^ (wild type) litter mates in Nissl-stained sections (Fig. 3a, b). In RAG1^−/−^ mice 24 h after TBI lesion volume was reduced (27.1 ± 4.1 mm^3^; 16.1 ± 2.4%-clh) by 17% compared to RAG1^+/+^ (32.4 ± 5.6 mm^3^; 19.4 ± 2.8%-clh; p < 0.05; Fig. 3a). Similarly, 5 days after TBI compared to RAG1^+/+^ (13.9 ± 1.0 mm^3^; 9.1 ± 0.7%-clh) lesion volume was reduced by 16% in RAG1^−/−^ (11.8 ± 1.2 mm^3^; 7.6 ± 0.7%-clh; p < 0.05; Fig. 3b).

#### Neurological impairment was not affected by RAG1 deficiency

Neurological Severity Score (after Tsenter^26^), assessed one day before and 1 and 5 days after CCI was not affected in RAG1^+/+^ compared to RAG1^−/−^ mice (before TBI: 0 ± 1 and 0 ± 1, 1 day after TBI: 5 ± 2* and 4 ± 2* and 5 days after TBI: 2 ± 2^**#**^ and 2 ± 1^**#**^, for RAG1^+/+^ and RAG1^−/−^, respectively, *p < 0.05 vs. before TBI and ^**#**^p < 0.05 vs. 1 day after TBI).

### Study C: effect of Neutrophil granulocyte depletion combined with AT1 inhibition

#### AT1 inhibition had no effect on neutrophil blood cell count

**Figure 4:**
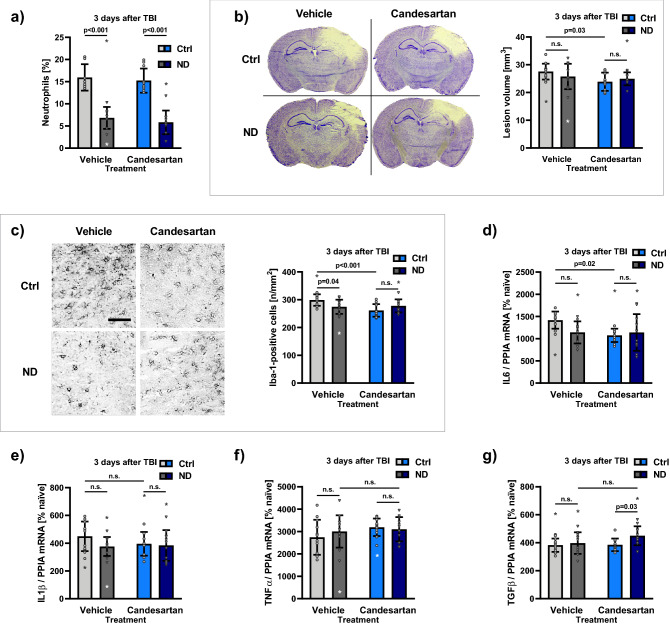
AT1 inhibition reduced brain damage and inflammation in control antibody treated mice while it had no effect in neutropenic mice (Study C). Male C57BL/6 mice were randomly allocated to four treatment groups: 24 h before, and 24 h after TBI, treatment with either anti-Ly6G for neutrophil depletion (ND) or IgG2a control antibody (Ctrl); then, 30 min after CCI (and then daily) additional treatment with candesartan (Cand) or vehicle solution (Veh). The Ctrl-groups are depicted in light colors: vehicle treated mice in silver-grey (Ctrl-Veh, n = 12), candesartan treated mice in blue (Ctrl-Cand, n = 12). The ND-groups are shown in dark colors: vehicle treated mice in dark grey (ND-Veh, n = 12), candesartan treated mice in dark blue (ND-Cand, n = 12). (**a**) Neutrophil granulocyte count [% WBC] at 72 h after TBI, i.e. 96 and 48 h after anti-Ly6G (ND) or Ctrl treatment, obtained with the ADVIA 2120i Hematology System. While anti-Ly6G significantly reduced neutrophils AT1 inhibition did not affect neutrophil count. (**b**) Lesion volume assessed 72 h after CCI in Nissl-stained sections: (**b**) left: representative pictures of Nissl-stained coronal sections at bregma level − 1.94 mm in the different treatment groups (Ctrl-Veh; Ctrl-Cand; ND-Veh; ND-Cand) at 72 h after TBI. (**b**) right: in Ctrl-Cand lesion volume was significantly reduced, compared to Ctrl-Veh treated mice. In anti-Ly6G treated mice AT1 inhibition had no effect on lesion volume. (**c**) Number of activated Iba-1 positive cells as a marker for microglia activation 3 days after TBI (in cortical perilesional tissue, ROI 0.52 × 0.65 mm^2^, bregma − 1.28 mm) and expressed as number of activated Iba-1 positive cells/mm^2^. (**c**) Left: representative immunohistochemistry images of Iba-1-stained sections taken from ipsilateral region of interest at bregma − 1.28 mm, adjacent to the lesion (scale bar: 100 µm), 72 h after TBI. (**c**) Right: neutrophil depletion reduced number of Iba-1-positive cells, compared to Ctrl in vehicle treated mice. In Ctrl-Cand treated mice activated microglia is significantly reduced, compared to Ctrl-Veh. (**d**) Gene expression of IL6 (real-time qPCR, normalized with PPIA, expressed as % naïve (0.06063 ± 0.01640 mRNA copies/PPIA)): IL6 was reduced by candesartan treatment in control antibody treated mice. (**e**) and (**f**) Gene expressions (normalized with PPIA, % naïve) of IL1β and of TNFα were not affected by any treatment at 72 h after TBI. (**g**) Gene expression of TGFβ (normalized with PPIA, % naïve) was elevated in ND-Cand, compared to Ctrl-Cand mice. Two-way analysis of variance (ANOVA) with Holm-Šidák’s multiple comparisons test. Data is presented as mean ± SD; p < 0.05. Excluded outlier values are presented with an asterisk (*).

To analyze an independent effect of candesartan treatment on neutrophil blood cell count we compared all treatment groups by two-way-ANOVA. While a sustained neutrophil depletion was achieved by anti-Ly6G (p < 0.001; Fig. 4a), AT1 inhibition did not affect neutrophil granulocyte count (Fig. 4a).

#### AT1 inhibition reduced lesion volume in control antibody treated mice, while it had no effect in neutrophil granulocyte depleted mice

Lesion volume was assessed 72 h after CCI in Nissl-stained sections (Fig. 4b). In candesartan and control antibody treated mice lesion volume (23.9 ± 3.3 mm^3^, i.e., 15.3 ± 1.8%-clh) was significantly reduced compared to vehicle and control antibody treated mice (lesion volume: 27.5 ± 2.9 mm^3^, 18.5 ± 1.6%-clh; p < 0.05; Fig. 4b). In anti-Ly6G treated mice, however, AT1 inhibition had no effect on lesion volume (ND-Veh: 25.8 ± 4.6 mm^3^, 16.4 ± 3.0%-clh compared to ND-Cand: 24.1 ± 2.4 mm^3^, 15.7 ± 2.2%-clh; Fig. 4b).

#### AT1 inhibition reduced cerebral inflammation in control antibody treated mice, while it had no effect in neutrophil granulocyte depleted mice

We quantified the number of perilesional Iba-1-positive cells as a marker for microglia activation 3 days after TBI (Fig. 4c) at bregma − 1.28 mm in cortical perilesional tissue in a ROI (0.52 × 0.65 mm^2^) adjacent to the injury (Fig. 4c). Results are expressed as number of Iba-1-positive cells/mm^2^. Compared to naïve (n = 80 ± 15/mm^2^), 72 h after TBI, there was a significant increase of Iba-1-positive cells in all groups (p < 0.001). In vehicle treated mice, the number of Iba-1-positive cells was reduced after administration of anti-Ly6G (ND-Veh: n = 275 ± 25/mm^2^), compared to Ctrl-Veh (n = 299 ± 21/mm^2^; p < 0.05; Fig. 4c). In control antibody and candesartan treated mice (Ctrl-Cand) number of Iba-1-positive cells was reduced (n = 262 ± 23/mm^2^) compared to Ctrl-Veh (p < 0.001; Fig. 4c). However, in neutrophil depleted mice AT1 inhibition had no effect on Iba-1-positive cells (ND-Cand: 277 ± 23/mm^2^).

Three days after TBI normalized gene expressions were assessed by qPCR, expressed as % normalized naïve expressions (IL1β: 0.00197 ± 0.00044, IL6: 0.06063 ± 0.01640, TNFα: 2.3488 ± 1.3114 × 10^–6^, TGFβ: 0.00560 ± 0.00078, iNOS: 0.00141 ± 0.00034 mRNA/PPIA). Expression of IL6 was reduced by candesartan treatment in control antibody treated mice (p < 0.05; Fig. 4d), whereas neutrophil depletion did not affect IL6 expression (Fig. 4d). Gene expressions of iNOS, IL1β and TNFα were not affected by any treatment (Fig. 4e, f). TGFβ was elevated in Cand-mice by anti-Ly6G-treatment (Fig. 4g).

#### Neutrophil depletion and AT1 inhibition had no effect on neurological outcome

Neurological assessment was performed 1 day before, and 24 and 72 h after TBI using a mNSS and time spent staying on the rotarod. Compared to pre-trauma values, CCI induced a significant impairment in all experimental groups 24 h after TBI in mNSS (p < 0.001). Time spent on the rotarod and mNSS improved over time 3 days after TBI without differences between the treatment groups.

### Study D: effect of RAG1 deficiency and AT1 inhibition

#### AT1 inhibition reduced lesion volume in RAG1-deficient mice

**Figure 5:**
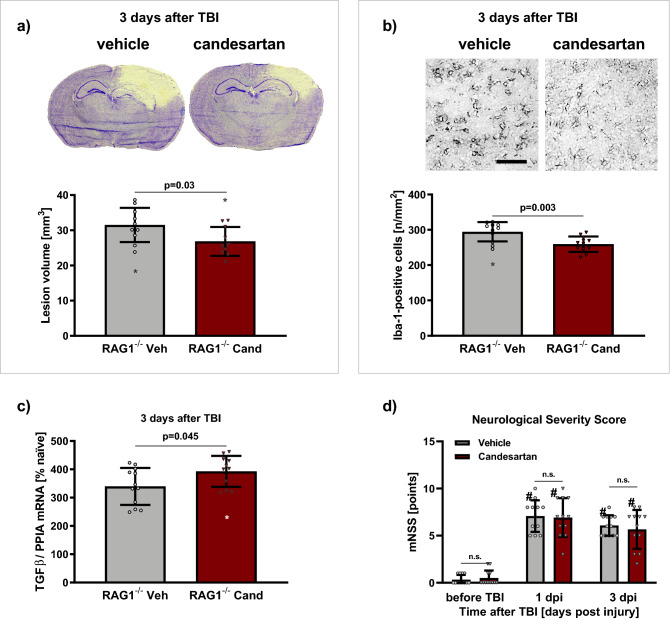
AT1 inhibition reduced brain damage and inflammation in lymphopenic RAG1 deficient mice (Study D). RAG1 deficient mice were randomly assigned to repeated vehicle solution (RAG1^−/−^-Veh, shown in grey) or candesartan treatment (RAG1^−/−^-Cand, depicted in red) at 30 min, 24 and 48 h after TBI (n = 12/group). As in study C, to facilitate comparability, we quantified the lesion volume, cytokine expression, and activated microglia 72 h after TBI. (**a**) Lesion volume was assessed in Nissl-stained brain sections three days after TBI. (**a**) Top: typical, representative pictures of Nissl-stained coronal sections at bregma level − 1.94 mm at 72 h after CCI. (**a**) Bottom: In RAG1^−/−^-Cand lesion volume was reduced compared to RAG1^−/−^-Veh. (**b**) Iba1-positive cells were counted in the cortical perilesional region of interest (ROI: 0.52 × 0.65 mm^2^, bregma − 1.28 mm) three days after TBI. (**b**) Top: representative immunohistochemistry images of Iba-1-stained sections taken from the ipsilateral ROI at bregma − 1.28 mm, adjacent to the lesion (scale bar: 100 µm) in vehicle and candesartan treated RAG1^−/−^ mice. (**b**) Bottom: 3 days after TBI AT1 inhibition (RAG1^−/−^ Cand) we observed reduced number of activated Iba-1 positive cells (/mm^2^) in the cortical perilesional ROI compared to RAG1^−/−^ Veh. (**c**) Normalized gene expression of TGFβ expressed as % naïve expression (0.00534 ± 0.00073 mRNA/PPIA; real-time qPCR) was higher in RAG1^−/−^-Cand compared to RAG1^−/−^-Veh. (**d**) Neurological outcome was assessed by the modified Neurological Severity Score (mNSS) at day 1 and 3 after TBI (dpi = days post injury). There was a significant increase of mNSS after TBI (^#^p < 0.05 compared to pre-TBI values), however, without any difference between the treatment groups. (**a**–**c**) Unpaired t-test with Welch’s correction. (**d**) Mixed effects analysis (REML) with Holm-Šidák’s multiple comparisons test. Data is presented as mean ± SD; p < 0.05; n.s. = not significant. Excluded outlier values are presented with an asterisk (*).

Three days after TBI in RAG1^−/−^ mice lesion volume was reduced by AT1 inhibition from 31.5 ± 4.9 mm^3^ (vehicle; 20.3 ± 2.7%-clh) to 26.8 ± 4.1 mm^3^ (candesartan; 17.0 ± 2.2%-clh; p < 0.05; Fig. 5a).

#### AT1 inhibition reduced cerebral inflammation in RAG1-deficient mice

Three days after TBI, AT1 inhibition reduced the number of Iba-1-positive cells in the cortical region of interest (0.52 × 0.65 mm^2^) adjacent to the lesion, from 295 ± 27 (n/mm^2^, vehicle treatment) to 259 ± 22 (n/mm^2^, candesartan treated mice; p < 0.05; Fig. 5b). Additionally, normalized gene expressions were assessed by qPCR, expressed as % naïve expression (IL1β: 0.00193 ± 0.00046, IL6: 0.06207 ± 0.01296, TNFα: 1.9017 ± 0.5769 × 10^–6^, TGFβ: 0.00534 ± 0.00073, iNOS: 0.00192 ± 0.00045 mRNA/PPIA). Expression of TGFβ was higher in candesartan treated compared to vehicle treated RAG1^−/−^ mice (Fig. 5c). However, at this time point gene expressions of inflammatory cytokines (IL1β, IL6, TNFα) and iNOS were not affected significantly by AT1 inhibition.

#### AT1 inhibition did not affect neurological outcome in RAG1-deficient mice

Neurological outcome was assessed by mNSS and rotarod at day 1 and 3 after TBI. There was a significant increase of neurological impairment (p < 0.001). However neurological deficit was not affected by candesartan treatment (Fig. 5d).

## Discussion

To investigate the yet unexplained role of cellular immune response in the context of AT1 inhibition following TBI, we examined the impact of candesartan treatment in both neutrophil-depleted and lymphopenic RAG1^−/−^ mice. The current findings indicate that both neutropenia and lymphopenia independently contributed to a reduction in brain damage following TBI. AT1 inhibition after TBI resulted in a decrease in brain damage and neuroinflammation in Ctrl mice with normal neutrophil counts, as well as in lymphopenic RAG1^−/−^ mice. However, in neutrophil-depleted mice, AT1 inhibition had no effect on brain damage or neuroinflammation. Hence, the current findings indicate that the neuroprotective effects of AT1 inhibition are partially mediated by neutrophils.

The experiments were conducted using our established CCI model^4,6,8,32,36,38,39^. We maintained standardized perioperative setting and conditions, to ensure stable physiological values^6^.

To deplete neutrophils, we employed a specific antibody against Ly6G, which selectively targets neutrophils while leaving other cell types unaffected^40–42^. Numerous dosage concepts exist for administering anti-Ly6G (4–40 µg/g). We based our dosage decision (app. 20 µ/g) on murine studies that used anti-Ly6G to achieve sustained neutrophil depletion^41,43,44^. To ensure a significant reduction of neutrophils, that was monitored using the WBC count, we conducted preliminary pilot dosage finding studies. We found that the present dosage (500 µg anti-Ly6G), and the application interval (24 h before and after CCI) were effective in sustainingly reducing neutrophils in the WBC count for the entire 3-day observation period after TBI^41,43,45^. Furthermore, this dosage did not affect other blood cell populations, animal survival, or body weight. In contrast, the widely used less specific anti-Gr1 (clone RB6-8C5) not only reduces Ly6G-specific cells (neutrophils, Gr1+/Ly6G+), but also other lines of WBC with Ly6-receptors (dendritic cells and subpopulations of monocytes and CD8 T-lymphocytes)^40,46^.

To examine the effects of lymphopenia in TBI, we employed RAG1 deficient mice^47,48^. While RAG1 plays a key role in VDJ-recombination and B and T cell differentiation, RAG1 deficient mice have small lymphatic organs without mature B and T lymphocytes^47–50^. Their lymphocyte differentiation is blocked at an immature stage^49^. In the present study, RAG1^−/−^ showed to have lymphopenia. Although, certain cell lines, such as NK cells, may be present in RAG1^−/−^^51^, the lymphocytes detected in the WBC count of RAG1^−/−^ mice are likely immature lymphocytes^49^.

The selected observation periods for each study were based on the optimal timing for the maximum infiltration of the two immune cell types into the brain tissue. Neutrophils, the dominant blood cell population 24 h after TBI^52,53^, infiltrate early, with parenchymal infiltration peaking at 1 day after TBI^18,19^. From the third day on after TBI, lymphocytes invade into cerebral tissue^6,18^. To enhance the comparability of the impact of candesartan between the neutropenic and lymphopenic mice, we opted to utilize an observation period of 72 h following TBI in studies C and D, respectively^18^.

To ensure timely accurate hematologic analyses and interpretation and to minimize pre-analytic errors a mouse-species-appropriate practical hematologic instrumentation was performed with consistent collection method from the retroorbital sinus and a validated automated veterinary analyzer according to our standardized protocol^54,55^. The quality showed to be adequate and results are consistent with recent data^54^. Neutrophil depletion with anti-Ly6G may not completely eliminate circulating neutrophils that lack Ly6G expression on their surface, as previously noted by Boivin et al.^56^. However, our flow cytometry analysis was performed independently of Ly6G, as we used the ADVIA 2120i Hematology system for differential blood cell count^29^. This is a well-standardized flow cytometry-based system that distinguishes and counts WBC through two methods: peroxidase, detecting MPO-positive cells, and lobularity/nuclear density, classifying cells by size, lobularity, and nuclear density. Both methods allow for accurate identification and quantification of WBC populations, including neutrophils. Therefore, we believe that the presented neutropenia by anti-Ly6G treatment is accurate.

In the present study, we observed a transient leukopenia as part of the inflammatory reaction following TBI, which we attribute to trauma-associated neutrophil sequestration^57^. In Ctrl treated mice WBC count normalized one day after TBI. Anti-Ly6G treatment resulted in a sustained reduction of WBC count in mice after TBI, lasting up to 3 days (within physiological range)^54^. Neutrophils, the most common granulocytes, generally comprise 20–30% of WBC count in naïve wild type mice (70–80% are lymphocytes)^54^. In Ctrl mice, TBI caused a shift from lymphocyte-dominated WBC to a sustained and significant increase in neutrophil count^52,53^. ND mice experienced neutropenia and leukopenia in comparison to naïve mice, with an increase in lymphocyte count from 24 h post-TBI onwards. RAG1^−/−^ mice appeared to be leukopenic, and lymphopenia was compensated by elevated neutrophils. AT1 inhibition had no effect on posttraumatic blood cell count in wild type, neutropenic and RAG1^−/−^ mice.

In the present study the selective AT1 blocker candesartan was chosen, that crosses the BBB^16^. Consistent with prior research^4,8,12,17^, a low dose of candesartan (0.1 mg/kg) was administered to avoid any negative impact on blood pressure, which can exacerbate TBI outcomes^58,59^. To achieve sustained AT1 inhibition, treatment was started 30 min after TBI and then repeated daily^8^.

The peritraumatic body weight, a surrogate parameter of well-being and intake of food and water, was not affected by any treatment. The behavioral and neuromotor functions are impaired in this acute phase but show quick recovery in all groups. However, we did not detect any significant treatment effects on neurologic outcome using the NSS which was originally developed by Tsenter et al.^26^ as a non-parametric assessment that focuses on motor function and behavior. Therefore, we tried to implement a more sensitive parameter for neurofunctional outcome, the Rotarod test, in subsequent studies. In studies C and D, we chose to combine the modified NSS (mNSS, adapted from Tsenter et al. 2008^4,26^) with the Rotarod test in an attempt to detect any treatment effects on both motor function and behavior. Unfortunately, despite our efforts, we were not able to demonstrate any significant treatment effects on neurofunctional outcome. The current neurological assessments may lack sensitivity to detect the effects under investigation, or the protective effects on brain tissue may not have been significant enough to improve neurological deficits.

During the first days after TBI, microglia/macrophages show high phagocytic activity to remove necrotic and apoptotic cells, whereas astrocytes start to form a scar-like barrier around the brain lesion^60^. This leads to a contraction of the lesion-surrounding tissue and decrease of lesion volume assessed with Nissl staining. This can be observed in the present study considering different lesion sizes at different post-TBI time points, with cavitation processes beginning later than 5 days after CCI in our model (for example, at 7 days after CCI)^27^.

Consistent with recent research, our study demonstrated that neutrophil depletion decreased lesion size at 24 h, and neuroinflammation, following TBI^61^. However, neutrophil depletion did not affect lesion size at 72 h post-CCI in our study. In contrast, a recent study demonstrated sustained reduction of brain damage up to 14 days after CCI by using a less specific Gr1-antibody to achieve neutrophil depletion^61^. We hypothesize that neutrophils may affect brain injury at a very early time point after injury, as supported by previous live-imaging observations in mice^62^, and our results on MPO mRNA expression. We examined the gene expression of MPO, which is widely recognized as a reliable neutrophil marker^37,63,64^. MPO mRNA expression was reduced in the injured brain tissue of ND (compared to Ctrl) mice at 4 h after TBI, that is 28 h after application of anti-Ly6G. However, at later time points (24 h after CCI, 48 h after anti-Ly6G application), MPO mRNA expression increased in ND mice to the level of Ctrl mice. Thus, a reduction was not detectable concerning neutrophil brain tissue infiltration, despite reduced neutrophils in the WBC count of ND mice. In the present study anti-Ly6G was applied 24 h before CCI, and in Study C also at 24 h after CCI. Based on our findings that MPO expression was not different between ND and Ctrl mice at 48 h after the application of anti-Ly6G, we believe that brain tissue neutrophil infiltration is only reduced by anti-Ly6G in the first 24 h after the application, and therefore, at later time points there is no effect of antibody-mediated neutrophil depletion on parenchymal neutrophil infiltration. This is supported by recent reports that indicate that antibodies against neutrophil Ly6G do not inhibit neutrophil recruitment^42^. Our findings suggest that reduced neutrophil infiltration into damaged brain tissue within the first few hours after TBI (rather than after 24 h) results in beneficial effects, including reduced TNFα expression at 4 h, reduced structural brain damage at 24 h, and decreased microglial activation at 72 h post CCI.

While T cells are known to play diverse roles in adaptive immune responses and inflammation regulation, their specific role in TBI pathogenesis remains unclear^65^. Recent findings suggest that cerebral infiltration of T cells exacerbates neuroinflammation without affecting lesion volume after TBI^66,67^. A previous study on closed-head injury found no significant differences in pathological or neurological parameters between wild-type and RAG1^−/−^ mice up to 7 days post-injury^47^. The authors concluded that adaptive immunity is not crucial for initiating and sustaining inflammatory neuropathology after closed-head injury^47^. Another TBI study by Fee et al. showed that CD4^+^ T lymphocytes contribute to the severity of the acute phase of TBI and that brain injury is attenuated in RAG1^−/−^ mice compared to wild-type animals^48^. Consistent with these findings, RAG1 deficiency in the present study led to reduced brain damage at 1 and 5 days post-TBI. However, at 24 h post-TBI, compared to wild type, neutrophil depletion was found to be more effective in reducing lesion volume (33%) than lymphopenia (17%). Despite this, the reduction of lesion volume by lymphopenia was found to be more sustained (up to 5 days post-TBI) than that of neutropenia (only 24 h after TBI). The acute post-traumatic cerebral infiltration of neutrophils is more pronounced than that of lymphocytes^18^. Hence, it is conceivable that reducing neutrophil infiltration may have a more potent anti-inflammatory effect in the initial 24 h post-TBI than reducing lymphocyte infiltration, possibly explaining why brain damage and inflammation are consistently reduced in studies involving neutrophil depletion^61^, while results are inconsistent in studies involving RAG1 deficiency^47,48^.

Emerging evidence indicates that the entire cellular immune response is modulated by the RAS^68,69^. AngII is a major mediator of cerebral inflammation and oxidative stress through AT1^7,70^. AT1 is widely expressed in the mature central nervous system, primarily in neurons, endothelial and smooth muscle cells, astrocytes, and microglia, which are important regulators of neuroinflammation^9,71^. AT1 is also expressed on migrating immune cells, like neutrophils, macrophages, and T-cells. AT1 activation triggers the production of chemokines, cytokines, and adhesion molecules, which promote the immigration of activated immune cells into the lesion site^7,72–75^. This process induces inflammation and generates high levels of ROS via NADPH oxidase activation. AT1 signaling modulates NADPH oxidase complex activity and promotes the transcription of pro-inflammatory cytokines through activation of NF-κB dependent transcription^15,76^. Subsequently, AT1 activation stimulates various kinases, which propagate inflammatory responses and apoptotic pathways^17,77–80^.

The present study demonstrated that repeated post-traumatic administration of candesartan in mice with normal post-traumatic neutrophil count led to reduced histological brain damage and decreased microglial activation at 3 days after TBI^8,12^. Candesartan treatment resulted in a 12% reduction in activated microglia/macrophages, while neutrophil depletion led to a 8% reduction, compared to vehicle and control antibody treatment. These findings are consistent with earlier studies where candesartan treatment reduced the number of neutrophils and microglia/macrophages 3 days after TBI^4,17^.

Recent studies have demonstrated that AT1 inhibition after TBI reduces cytokine expression^7,8,81^. The pleiotropic cytokine TNFα is implicated in BBB dysfunction and the transmigration of WBC into brain tissue. It induces neuronal loss via microglial activation^2,82–84^. After an early upregulation in the first 8 h after TBI^83^ TNFα decreases significantly in the following time^85^. This kinetic could explain that in the present study there is only a reduction of TNFα 4 h after TBI in ND mice. Cerebral IL1β expression increases in the first hour after TBI and reaches highest levels 12 and 24 h after experimental TBI^86^. Reduced activity of IL1β showed improved neurological outcomes and reduced infiltration of neutrophils^83,87–89^. The absence of an effect on IL1β expression in the present study following neutropenia, RAG1 deficiency, or AT1 inhibition may be attributed to low levels of cytokine mRNA at 3 days post CCI^15^. Numerous studies have reported upregulation of IL6 following TBI, which is associated with increased microglial activation and neurological impairment^2,90^. Recent clinical studies suggest a correlation between elevated serum levels of IL6, increased ICP, and severity of TBI^91^. IL6 has also been shown to regulate the migration of neutrophils during acute inflammation^92^. In this study, AT1 inhibition led to a reduction in IL6 expression three days after TBI in Ctrl mice. However, candesartan did not affect IL6 expression in neutropenic mice.

Limitations of the present study are the short duration of reduced neutrophil infiltration into brain tissue, only detected at 4 h after CCI, and not at later time-points, as well as the absence of observed effects on neurological outcome. The fact, that we were not able to demonstrate the direct effect of AT1 inhibition on neutrophil infiltration into the brain tissue is a further limitation. Another significant limitation of the current study is the exclusive focus on acute post-traumatic time points. Further investigation is needed to examine the long-term impact of AT1 inhibition on structural brain damage, chronic brain inflammation, and cognitive dysfunction.

In a recent study, the protective effect of post-traumatic AT1 inhibition in young adult and aged mice was attributed to a decrease in microglia activation and an increase in anti-inflammatory microglia polarization. One major finding of the study was a significant reduction in neutrophil infiltration^4^. AT1 is expressed on both circulating neutrophils and lymphocytes^74,93^. However, in the previous study, perilesional T-cell immigration was not affected by AT1 inhibition^4^. The neuroprotective mechanisms of AT1 inhibition in the acute phase after TBI may thus not depend on the adaptive lymphocyte response. A recent study showed that expression of CD62L on human neutrophils is modulated by AT1 receptors, on various pathways involving ERK1/2, MAPK, and calcineurin^94^, leading to reduced transmigration of neutrophils. It has been shown that AT1 inhibition leads to down regulation of important recruitment proteins like ICAM1 in endothelial cells, and CD11b/CD18 on WBC, and reduces post traumatic increase of BBB permeability. Consequently, AT1 inhibition leads to a significant reduction in the infiltration of immune cells^95–98^. A recent murine cerebral transcriptomic analysis after TBI showed strong alterations of gene transcription, particularly of the innate immune response, by candesartan treatment^99^. Therefore, AT1 inhibition may have a direct and modulating anti-inflammatory effect on invading neutrophils and resident activated microglia^17^. AT1 inhibition appears to provide neuroprotection by reducing the inflammatory response of the innate immune system, as evidenced by reduced microglial activation and decreased infiltration of neutrophils. This suggests that the protective effect of AT1 inhibition is mediated by its anti-inflammatory properties^4^.

## Conclusion

The present study indicates that the reduction of immune cells in both the innate and adaptive immune system, specifically neutropenia (ND), and lymphopenia (RAG1^−/−^), independently leads to decreased brain damage after TBI. Moreover, the study demonstrates that posttraumatic AT1 inhibition decreases brain damage and neuroinflammation in mice with normal neutrophil counts and in lymphopenic mice. However, in neutrophil-depleted mice, AT1 blockage had no effect on brain damage and neuroinflammation. We conclude that the neuroprotective effects of AT1 inhibition are independent of lymphocytes but dependent on neutrophils. Thus, reduced neutrophil invasion into the injured brain tissue may mediate neuroprotection by AT1 inhibition. In summary, the study highlights the importance of immune cells in mediating neuroinflammation after TBI and the potential of AT1 inhibition as a therapeutic strategy against exacerbated neuroinflammation following TBI.

## Data Availability

The datasets generated during and/or analyzed during the current study are available from the corresponding author on reasonable request.

## Abbreviations

AngII: Angiotensin II
AT1: Angiotensin II receptor type 1
Cand: Candesartan treated mice
CCI: Controlled cortical impact
%-clh: Percentage of contralateral hemisphere
Ctrl: Control antibody treated mice (IgG2a)
ICP: Intracranial pressure
IL1β: Interleukin 1β
IL6: Interleukin 6
iNOS: Inducible nitric oxide synthase
MPO: Myeloperoxidase
ND: Neutrophil depleted mice (Ly6G-antibody)
PPIA: Cyclophilin A
qPCR: Quantitative polymerase chain reaction
RAG1: Recombination activating gene 1
TBI: Traumatic brain injury
TGFβ: Transforming growth factor β
TNFα: Tumor necrosis factor α
Veh: Vehicle solution treated mice
WBC: White blood cells
